# The bright side of digitization: Assessing the impact of mobile phone domestication on left-behind children in China's rural migrant families

**DOI:** 10.3389/fpsyg.2022.1003379

**Published:** 2022-10-19

**Authors:** Jiamei Tang, Ke Wang, Yuming Luo

**Affiliations:** School of Journalism and Communication, Guangdong University of Foreign Studies, Guangzhou, China

**Keywords:** digitization, left-behind children, migrant family, mobile phone, rural China, separation

## Abstract

This study examines the mobile phone practices of rural left-behind children (LBC) whose one or both parents migrate to cities for better earnings and the impact of such practices on migrant families in China. The study has used ethnographic approach by conducting participant observations and interviews of 21 LBC, residing in Guangren village, south China's Guangxi Autonomous Region. The study uses domestication theory to analyze these LBC's adoption of mobile phones in their daily routines and spaces in and out of their households. The key findings are as follows: (a) the LBC used mobile phones primarily to engage with their distant parent(s); (b) through collaborative efforts, they tried to enhance familial connections; and (c) they overcome the separation issue by co-participating in ongoing events, thus making the domestication of mobile phone a distant solving of real-world problems faced by migrant parent(s) and their LBC. The study concludes that LBC's innovative uses of mobile phones empowered them by building shared virtual space with their migrant parent(s), *via* which they handled the separation issue. In such shared virtual spaces, LBC's families have developed rich expressions of familial connections in various forms based on the limited perpetuate connectedness.

## Introduction

The relationship six among migration, internal or external, and the connectedness to families and culture through various modes of communications has remained an important discussion among the scholars (Seymour and Walsh, 2013; Koylu et al., 2014; Shah et al., 2019; Telve, 2019; Liu et al., 2020a; Moran, 2022). In contrast to the argument of networked families (Rainie and Wellman, 2012) which highlights the impact of digitization of information and communication technologies (ICTs) on the structure and organization of families in general, ICTs are considered vital for migrant families, especially those out of “forced” migration because of economic incentives (Meyers and Rugunanan, 2020). ICTs are used to maintain the operations of migrant families and familial connections and support that are important for the wellbeing of all family members, which contributes to the wellbeing of the society in general (Pearce et al., 2013). Some research approaches this issue from parenting or mothering perspective and argues the usefulness, if not indispensability, of ICTs on migrant parents or mothers' conducting of care and support to their left-behind children (Hondagneu-Sotelo and Avila, 1997; Madianou and Miller, 2011). Some other researchers examine how the technological affordances of digital ICTs have brought new forms of connected migrant families such as transconnectivity (King-O'Riain, 2015). Existing literature takes an adult position to examine the maintenance of separated families by either migrant parent(s) or left-behind adults and pays no attention to the role of children in the maintenance of migrant families. In the age of digital ICTs, some children are their parents' technological experts, and a parent–child role reversal has been found in terms of the adoptions of digital devices (Wang, 2020). Therefore, children's contributions to the operations of separated migrant families should not be ignored. We take an initial attempt in this research direction. Besides, most literature focuses on international labor migration and the maintenance of familial connections cross border and culture. Internal migration as a context of separated families has been insufficiently explored.

The issue of family separations out of internal labor migration is especially relevant to China. The large internal migration population moving from rural areas to cities for better economic income has been one of the boosters to China's high-speed economic growth in the past three decades. The wellbeing of the migrant family, particularly the wellbeing of the children left behind by the migrant labor force, is a challenging issue for the government and a widespread social concern. Wang and Zheng (2022) argued that many rural LBC suffer from emotional dilemmas because of the lack of parental company and emotional interaction, and therefore, they seek emotional compensations from the uses of social media platforms, online entertainment, and online gaming that are enabled by their mobile phones. Such entertainment uses of mobile phones become the major ways for LBC and other rural adolescents to engage in socialization (Guan et al., 2020) and emotional socialization (Wang and Zheng, 2022) in digital age. However, heavy uses of the entertainment function of the mobile phones result in LBC's addictions to mobile phones (Cai et al., 2021). Current research on the relationship between digital ICTs and LBC of rural migrant families concentrates on the impact of high penetration rate of mobile phones on LBC alone. This research fills a gap by examining LBC's uses of mobile phones for familial connections and its impact on rural migrant families. In rare research on parent–child communication in migrant families, Hu and Mao (2019) explored co-presences achieved *via* mobile phone communication between LBC and their migrant parents and found that phatic co-presence on mobile phone communication is mostly seen between LBC and their migrant parent(s). The authors proposed that both long distance and ICTs limited intimate parent–child communication in rural migrant families and provided breakthroughs for creating tighter parent–child communication (Hu and Mao (2019), p. 68). This research fills a gap by examining the various ways that LBC use mobile phones in addressing separation with their migrant parent(s) including both co-present and asynchronous means.

This research offers a theoretical contribution to the studies on the relationship between ICTs and LBC problem in China by applying domestication theory to find out how LBC negotiates with mobile phones in their daily life. We went into the life world of LBC to see the context of their mobile phone uses and get their narratives on their uses. What we explored is not how mobile phones impact rural migrant families but how LBC explored mobile phones in the temporal-spatial contexts of their daily life to get connected with their distant parent(s). We look at the social practices of LBC around the mobile phones instead of the technological affordances *per se*. Therefore, we ask this research question: how do LBC adopt mobile phones to cope with their separation with parent(s) and with what effect on LBC families?

The paper is organized as follows. The Background section frames the contexts of this research including the origin of the rural migrant workers and LBC and the prevalence of mobile phones in contemporary China. Next, relevant literature is reviewed to demonstrate gaps of this research in greater details. In the following section, the methodology is explained, followed by the Findings and Analysis section. In the end, the Conclusion section theorizes the findings and presents limitations and future research directions.

## Background of the study

China's economic boom since the adoption of Reform and Opening-up Policy in 1978 has been accompanied with an uneven development between eastern coastal and inland regions, and the rural and urban areas (Mu and Hu, 2016). The gaps of economic development and income have been the major factors contributing to internal migration from the rural to the urban since 1980's. The scale of rural-to-urban migrant workers for economic gains has increased annually from 137 million in 2017 (To et al., 2020) to almost 300 million in 2021, according to the National Report on Migrant Worker Monitoring and Survey 2021 released by National Statistical Bureau in 2022 (National Statistical Bureau, 2021), or one out of every five Chinese. Most migrant workers are working in labor-intensive and/or low-wage sectors such as manufacturing, construction, wholesale and retail, and service sectors, sometimes with poor working conditions and long working hours (Mu and Hu, 2016). Though migrant workers living and working in cities, some for years, have become the major component of labor-intensive workforce in cities, they have been excluded from equal treatment as their urban counterparts because of *Hukou*-based social exclusions (Zhang et al., 2014; Liu and Erwin, 2015). *Hukou*, or Chinese household registration, “is a residence permit linked with political and economic rights” (Zhang et al., 2014, p. 1441). Rural residents and their children are not entitled to social welfare, health care, public school education, etc. in cities in spite of the fact that they work, live, or are born in urban areas (To et al., 2020). Therefore, many rural-to-urban migrant workers have to leave their children at rural home, creating a large number of left-behind children in China. According to Ministry of Civil Affairs' data on rural left-behind children released in 2018 (Ministry of Civil Affairs Chart, 2018), there were about seven million left-behind children (LBC) in 2018, 22% off from the nine million figures in 2016. Many migrant workers go home only one time every 1 or 2 years (Pan et al., 2013), leaving LBC in the care of grandparents or other relatives.

In addition to the phenomena of rural-to-urban migrant workers and LBC, another context of this study is the prevalence of mobile phones in China. Fortunati et al. (2010) pointed out that mobile phones diffused quickly in China which leads the world in ICT production and becomes the primary market of mobile phones, accounting for one-fifth of global mobile phone subscriptions before 2010 (Liu et al., 2012). Mobile phone penetration rate continued to increase annually from 2013, reaching 78.7% in 2019 (Mobile Phone Penetration in China, 2013–2019). Till mid-2022, mobile phone subscribers reached 1.668 billion (China Internet Network Information Center, 2022b), indicating that some population owns more than one mobile phone. With the high penetration rate of mobile phones, rural China has been impacted by “mobile revolution” (Rainie and Wellman, 2012) owing to the government's vigorous efforts in building rural information infrastructure (Leng, 2022).

Mobile phones become the dominant Internet devices in China. Among China's 1.51 billion Internet users, 1.47 billion got online *via* their mobile phones. That is, 99.6% of online surfers accessed the Internet using their mobile phones, whereas only one-third online population used desktop computers, the second most-used Internet device (China Internet Network Information Center, 2022b). Mobile phones are also important for Internet access in rural China. Up to the end of 2015, more than 87% rural Internet users got online with mobile phones (China Internet Network Information Center, 2016). This rate is even higher in under-18 age group in rural China and reaches 92.7%in the end of 2021 (China Internet Network Information Center, 2022a). By mid-2022, rural Internet users reached 293 million, penetrating 58.8% of China's rural regions and accounting for 27.9% of China's online population (China Internet Network Information Center, 2022b).

## Literature review

### Left-behind children and the social concern

The issue of left-behind children is widely explored in Chinese context. Gu (2022) pointed out that the miserable plight concerning the life and wellbeing of LBC is well-documented by mainstream newspaper articles, policy documents, and large scholarship in and outside of China since the early 2000's. Studies found out that left-behind experiences, described as “the feelings of loneliness and abandonment that children could experience if their parents had migrated without them” (Murphy, 2022; p. 188), have negative impact on children as young as preschool kid (Hu et al., 2020) and the negative impact could long last until the LBC reach the college level (Liu et al., 2020a; Xie et al., 2021).

Previous studies highlighted the concerns of the negative impact of left-behind experience on LBC's psychological health. Studies reported high rates of psychological depression and anxiety of LBC (Cheng and Sun, 2014; Lu et al., 2020) compared to those children who are living with their parents. A study that is based on 1,500 personal narrative texts of LBC on the Chinese social media platform Zhihu reported that LBC show higher risk of depression and suicide compared to the children who are living with their parents (Lyu et al., 2022). Left-behind experience in children becomes childhood trauma that can lead to higher suicide risk and even can impact the LBC college going children (Xie et al., 2021). The extension of left-behind experience into adulthood can be a significant risk factor for mental health problems (Liu et al., 2020b). Research reveals that LBC suffer more stressful life events, such as depression (Tang et al., 2018; Lan et al., 2019), non-suicidal self-injury (Lan et al., 2019), unintentional injury (Hu et al., 2018), and poorer psychological resilience (Zhou et al., 2021). Dai and Chu (2018) showed that LBC have lower level of happiness and higher level of anxiety than non-LBC.

Besides psychological disorders, LBC are found to exhibit behavioral disorders (Ge et al., 2019), including lower levels of esteem (Tang et al., 2018; Yu et al., 2022), poorer physical health (Jin et al., 2020), lower academic performance (Hu et al., 2020), more aggressive behavior (Yu et al., 2022), and worse off cognitive development (Lu et al., 2020). A research on rural LBC based on the subjective discourses of the LBC reveals that their rights to provision, protection, and participation are ignored, which have a negative impact on their development (Zhao, 2018).

In addition to the political-economic factors at institutional level underlying LBC's plight, which is arguably not fully discussed according to Gu (2022), the psychological, cognitive, and behavioral problems found in LBC are arguably caused by child–parent separation (Ge et al., 2019), or “pathological” family life (Gu, 2022). With the prevalence of mobile phones in China today and the argued affordances of the mobile phone technology, such as contactability anytime anywhere (Green and Haddon, 2009), the carrying of Internet on mobile communication (Silva and Shella, 2014), the anywhere anyplace connectivity (Vanden Abeele et al., 2018), and mobile phone at the forefront of digital convergent media (Goggin and Hjorth, 2014; p. 2) with multimodalities because of numerous downloaded applications other than native applications of mobile phone (Reuver et al., 2016), it is worthwhile to make an updated examination of the relevance of mobile phones to the address of the LBC problem of parent–child separation or the alleviation of LBC plight. As the literature on the relationship between ICT and transnational migrant families demonstrates, mobile phone uses have the potential to maintain transnational familial connectedness.

### Migrant families, mobile phone usages, and the connectedness

There is rich scholarship focusing on the uses of ICTs and its role in connecting the transnational migrants (Demirsu, 2022), transnational families (Hoang and Yeoh, 2012; King-O'Riain, 2015; Pajnik, 2015; Chen, 2022), and transnational mothering (Madianou and Miller, 2011; Peng and Wong, 2013, 2016; Chib et al., 2014; Meyers and Rugunanan, 2020). These studies highlighted the usefulness of mobile phones in maintaining familial ties in migrant families by overcoming temporal-spatial-socio-separation, thus providing virtual presence of migrant parent(s) or co-presence to their left-behind children. Madianou (2014) argued that as the communication tools, such as smartphones, are becoming cheaper, the ICTs are gaining importance in transnational family relations. Mobile phone usages are no more limited to calling and text messages, rather they extended to multimodality means of communication, such as connecting the users with their transnational family members through social media platforms. For example, Skype and Facebook are the key social platforms in western societies that are connecting family members and giving them a feeling of homely experience for both the left-behind family member and the migrant ones (Pajnik, 2015). King-O'Riain (2015) also argued that, using Skype, transnational families practice simultaneous and ongoing belonging and create spaces of transconnectivity across distance. Live video calls mediated by mobile phones have added a new spatial dimension to the existing temporal dimension of everyday co-presence by allowing for greater room of self-expression and bonding in the form of visual performance, spatial sharing, and spatial-temporal longing between intergenerational family members of transnational migrants (Demirsu, 2022).

The role of transnational mothering is another serious concern that has been debated by the scholars as their role is crucial to connect well their LBC and to avoid LBC's serious psychological issues, such as anxiety, depression, and loneliness. Madianou and Miller (2011) argued that Philippine government and telecommunication agencies are aware of the social cost of left-behind family members, thus empowering the mothers by providing them with the cheapest ways to reconnect with their children, so that the UK-based Filipino migrant mothers can play their constructive role as parents. Peng and Wong (2013) highlighted that frequent communication between migrant mothers and their children on mobile phones is migrant mother's active participation in children's daily lives, which creates virtual presence. Chib et al. (2014) argued that mobile phone has an indispensable role in bridging the distance between intensive mothering and their LBC through voice and text messages and through cyberspaces, thus redefining the migrant mothers' identities and familial relationships in digital era. For Somali mothers working in South Africa, they use mobile phones for distant parenting, including transferring remittances to their children's caretakers, sustaining emotional bonds, teaching religious beliefs, and encouraging their children's educational pursuits (Meyers and Rugunanan, 2020). Besides communicating with their children, transnational mothers also use mobile phones to communicate with various caregivers, including the left-behind spouse, grandparents, siblings, and other family kins for sharing and collaborating of children-caring (Peng and Wong, 2016).

Most of the studies on ICTs' role in maintaining transnational families focus on the migrant perspective and only few are on the left-behind perspective. Pearce et al. (2013) examined the left-behind Armenian family members of labor migrants and suggested ICTs counter the physical fragmentation of the family and ease the strain of family separation by allowing distant participation in day-to-day life, that is, creating “a virtual, yet physical, integration of absent family members” (p. 2149). Chen (2022) found that despite long-distance separation, left-behind women use various social media applications on mobile phones which are playing critical role to reunite the family members to coordinate well on child-caring and maintain the family relationships. In other words, digital media has minimized the geographical distance among the migrated family members, thus allowing them to collaborate and to play their due role to look after LBC. Madianou and Miller (2011) provided rare accounts of LBC and found that not all children react positively to their migrant mother's transnational parenting on mobile phones, accusing of, for example, transnational calls irritating or intruding. However, there is no study that can explain how children participate in transnational parenting. These previous studies are mainly focusing on how the LBC are reacting to digital media usage by their migrant parents. Therefore, it provides a rationale for this study to fill the gap and evaluate the role of emerging digital and convergent media for migrant families from the perspective of LBC.

The previous studies are mainly focusing on the context of international migration. Rare studies that focus on internal migration and parents' connectivity with their left-behind children are available. At that backdrop, this study bridges the gap and analyzes LBC's uses of new digital technologies (mobile phones) and their efforts to coordinate with their migrant parents. In addition, this study will also contribute to the existing literature on LBC and the internal migration in Chinese context.

### The use of mobile phones in LBC families

Owing to the high penetration rate of mobile phone in rural China, mobile phones are found popular with LBC (Li et al., 2019; Guan et al., 2020). Yet, the use of mobile phones between migrant parents and their LBC has been a mixed blessing in media discourses since the diffusion of mobile phone in China (Xu, 2016). Research indicates that migrant parents rely heavily on mobile phones to communicate with their LBC for the maintenance of quality parent–child relationships (Pan et al., 2013; Liu and Leung, 2017; Gan et al., 2020). Liang et al. (2022) argued that the phenomenon of mobile phone parenting is prevalent in rural migrant families. For migrant mothers, mobile phone communication with their LBC is important to pillar their self-evaluation of maternal identity (To et al., 2020). Mobile phone screens *via* video calls become places for LBC to meet their distant parent (Gan et al., 2020). Yet, phone calls made on the mobile phones are found insufficient in terms of frequency of calls and depth of conversations for LBC to eliminate the feeling of alienation toward their distant parent(s) (Pan et al., 2013). Hu and Mao (2019) confirmed that long distance and gaps of media literacy between migrant parents and LBC contribute to the lack of communication between them and mobile phone communication has not become the driving force between parent–child communication and relationship maintenance in rural migrant families. Moreover, while parents allow their children to use mobile phones for the convenience of contact, most children use mobile phones primarily for entertainment purposes (Li et al., 2019). Cai et al. (2021) reported that LBC's Internet addiction is two times as high as those of non-LBC. The key argument is that as parental migration leaves the children unchecked, their Internet addiction is higher than those of non-LBC on whom parents have the supervision. As a result, LBC have high risks of mobile phone dependency (Guan et al., 2020), thus negatively affecting the LBC's life events as well as their academic engagements, such as problems with academic persistence and academic procrastination (Zhen et al., 2020).

The literature demonstrates an obvious gap between LBC and their parents in uses of mobile phones. While migrant parents enjoy the convenience of mobile phones for perceivably satisfactory or unsatisfactory parenting, what role their children play in such distant parenting is not researched. The other gap is the lack of in-depth exploration on LBC's practices of various mobile phone affordances on communicating with their distant parent(s) in addition to synchronous communication. Besides, existing scholarship pays less attention to the ways that mobile phones are useful for LBC families including both parents and children. Therefore, this study is an effort to address the following two-pronged questions: (a) how do LBC adopt mobile phones to cope with their separation with parent(s), and (b) with what effect on LBC families? We have applied domestication theory to look at in-depth of the issue of LBC and their usage of mobile phones in the internal migration process to minimize the social cost and to ensure the better psychological health care of LBC.

### Domestication theory

Domestication theory is a process of taming or bringing under control to be a member of the household by adopting new technologies in the social process (Silverstone, 1994; Berker et al., 2006). It is also used in understanding the role of ICTs in the relationship between private households and public worlds (Silverstone et al., 1992). Taking the household as the site to examine the social, cultural, or political significance of private uses of ICTs, domestication theory proposes four correlating processes in such examination: appropriation, objectification, incorporation, and conversion (Silverstone et al., 1992; Silverstone, 1994; Haddon, 2006, 2007). Whereas, appropriation refers to the purchase of ICT products as commodities into the household, objectification is about the spatial location of the products in household, incorporation means the embeddedness of ICTs into daily routines, and conversion means the articulation of the private sphere with the public out of the consumption of ICT products (Silverstone et al., 1992; Haddon, 2006, 2007, 2011; Silverstone, 2006).

Domestication theory has been applied widely in studies on media and technologies. It dissolves the wrestles for causality in terms of contributions to social change between the accused technological determinism, or the important role technological advancement plays in social development and change, and social construction of technology, emphasizing human's agency in creating and applying technology as well as circumstances in such creation and application and technological affordances *per se* (Baym, 2015) by looking at the uses of ICT as the processes of negotiations between ICT objects and their users (Berker et al., 2006). Domestication theory provides a perspective to look at the daily uses of ICTs in the household through which to see how daily life practices are related to bigger social processes and structures (Berker et al., 2006). With its emphasis on daily practices of ICT's uses, domestication theory provides not only theoretical perspective and conceptual tools to study the uses of ICTs, but also methodologies with an emphasis on in-depth empirical methods (Berker et al., 2006). For example, objectification and incorporation point to the timing and spaces of ICT uses, and conversion includes how technologies are talked about and displayed (Haddon, 2006). Moreover, domestication theory provides rich resources and flexibilities for extending morphosis of the original concepts. Negotiations between ICTs and households, for example, extend to involve the household's cognition, evaluations, aesthetics, values, norms, and every day practices (Hartmann, 2006), as well as parents' beliefs (Willett, 2017). The concept of re-domestication is developed, arguing the shift of meanings, roles, and uses of old technologies when new technologies are adopted (Grošelj, 2021), which is widely seen in mobile phone as a convergent media (Goggin and Hjorth, 2014). The theory has also been applied beyond the restrictions of households into, for example, children's education center and the uses of ICTs for children there (Martínez and Olsson, 2021).

This research uses domestication theory as theoretical framework to look in-depth at the LBC's uses of mobile phones to connect with their migrant parent(s) and beyond. We used four concepts, that is, appropriation, objectification, incorporation, and conversion to see when and why our respondents were allowed to use or own mobile phones, when and where they used their mobile phones, what they did with their mobile phones in those routines and spaces, and what cognition, evaluations, values, norms, every day practices (Hartmann, 2006), and parents' beliefs (Willett, 2017) were reflected from such uses. Meanwhile, we take full use of the flexibility of the theory and take account of the portability of mobile phone, individualization trend in the use of mobile phone and mobile apps as non-tangible objects (Matassi et al., 2019). We have used ethnographical approach to achieve the in-depth data with details and insights, which is also widely seen in domestication research.

## Methodology

### Field visit: Location and its key characteristics

The study is based on the analysis of semi-structured interviews and participant observations from fieldwork in a village in Guangxi Zhuang Autonomous Region in south China. In existing research on LBC in China, Guangxi as one of the major sources of migrant workers in neighboring manufacturing centers such as Guangzhou and Shenzhen has not been researched yet. One of the authors comes from a town in the most populous county-level city in Guangxi. To take advantage of this author's dialect and local knowledge, Guangren village, the largest village affiliated to his hometown, was chosen as the site of the ethnographic fieldwork. Because of financial constraints, only one author conducted field visits to the site, which is 5-min drive from his hometown, between June 2021 and March 2022. According to the village committee, Guangren village had a registered population of more than thirteen thousand including those leaving the village for working in cities at the time of the fieldwork. More than 10% of Guangren population is children. The village has three kindergartens and one primary school, though secondary and high schools are in the nearby town that offer boarding facilities. As the village is nearby Guangzhou and Shenzhen, two of the most popular destinations for internal migrant workers, many villagers try to find better-paid jobs in these two cities and therefore leaving their children and family behind.

### Sampling

The study has used stratified random sampling method in line with Wu (2021) findings in four Chinese provinces that around 10% of LBC are pre-teens whereas roughly 85% are those in the 11–15 age group, and the rest of LBC are 16–17 age group. We sampled among three strata covering primary, secondary, and high school LBC in Guangren village. One of the authors finished his high school education in the town to which Guangren village is attached and therefore used his local social capital and network to get the recommendations of potential respondents and their caregivers. This author contacted and talked with many recommended caregivers, parents, and teachers before the selection of samples. In the end, 21 LBC were recruited in the considerations of the varieties of these respondents in terms of age, gender, economic background, and family structure. This author gained double consent both from 21 respondents and their parents and caregivers. The samples include four primary school LBC, 14 secondary school respondents from 12 to 16, and three high school LBC, echoing Wu (2021) findings on age group distribution of LBC. The demographic structures of these 21 respondents vary (refer to the Appendix). Most respondents lived in extended families with grandparents, their adult children, and sometimes other grandchildren as well. The economic conditions of our respondents' households varied with most at average level.

### Interviewing, participant observations, and the respondents' identity

After the samples were selected, one of the authors made visits to 18 families to chat with the respondents and their caregivers and took part in their farm work such as fish catching and fruit picking to gain their trust. Home visits immersed one of the authors in the daily lives of the respondents, so that he observed the uses of mobile phones by respondents at home. The sites of participant observations also include public areas in the village such as the farm lands, boba tea café, snack food stalls, KTV, and basketball court, when respondents used mobile phones outside home. In addition to participant observations, semi-structured interviews were conducted with all 21 respondents and some of their migrant parents and kin caregivers. The purposes of in-depth interviews are to listen to respondents' narratives of their uses of mobile phones and to distinguish how they used mobile phones to address the separation from their migrant parent(s). Each interview lasted about half an hour to generate in-depth data of LBC's uses of mobile phones and meanwhile not to exhaust respondents. The interviews were conducted in local dialect by one of the authors and recorded for him to do the transcriptions in Chinese. In total, two authors read the transcripts repeatedly to sift findings relevant to the research question and one author read those findings repeatedly to seize emerging themes without using coding book.

To protect the respondents' identity, we used coding system in which a number indicates the order of interview while a letter indicates the gender of the interviewee. F refers to female and M refers to male. The profiles of the respondents are indicated in the Appendix.

## Analysis and the key findings

The results indicate that mobile phones are popular among adults in Guangren Village. Almost all the adult caregivers of our respondents had their own mobile phones, including the grandparents. Therefore, our respondents accessed mobile phones at quite early age. LBC usually initiated the discussions with their parents on owning a mobile phone before they moved to middle school with good reasons for the necessity to keep a mobile phone. Analysis shows that educational value, needs to communicate with parents, and convenience of online transaction are some of the key reasons that all our teenage respondents claimed to convince their parents for allowing them to possess a mobile phone, although LBC had their own actual reasons to have a mobile phone, which is common among adolescents (Oksman and Turtiainen, 2004).

According to mostly the narratives of our respondents in the interviews and the observations in the ethnographic fieldwork, the typical uses of mobile phones, such as the descriptions of Internet users as embodied, embedded, everywhere (Hine, 2015), emerged in the teenage respondents' stories of their mobile phone uses on weekends and holidays mainly, as they were not allowed to use mobile phones at school. We found that they made initiative and active uses of mobile phone affordances to satisfy their communication needs with their migrant parent(s). They demonstrated more digital competence, the knowledge of a variety of mobile phone functions and apps and the capacities to use them skillfully than their parents and adult caregivers. Their digital competence helps them alleviate their longings for distant parent(s) and combine various mobile phone functions and apps to keep connected with their distant parent(s). Though each respondent had his or her own unique demonstration of digital competence, all LBC and their migrant parent(s) made collaborative efforts. In this regard, LBC have sometimes played the leading role to perpetuate familial connections as means to overcome temporal-spatial separation. LBC engaged with their distant parent(s) by activating regular or even constant contact, asking their migrant parent(s) to solve practical problems, and creating virtual accompany with the help of the contactability anytime (Green and Haddon, 2009), connectivity anywhere (Vanden Abeele et al., 2018), and multimodality afforded by mobile phone technologies.

### LBC's initiatives and proactiveness

Keeping in direct contact, or instantaneous communication, is one of the motivations for migrant workers to use mobile phones to assist in distance parenting and build quality parent–child relationship and interaction in migrant families (Liu and Leung, 2017; To et al., 2020). Previous studies focus on the parents' perspective, usually migrant mothers, on their agencies of using ICTs to participate in their LBC's life (Peng and Wong, 2013). This undertaken study provides a LBC's perspective with their initiative, innovative, and various practices of mobile phones, highlighting that the contributions LBC made proactively to address the separation problems they confronted. In our respondents' narratives, keeping in contact with migrant parent(s) was a take-for-granted use of mobile phones. They used words such as “natural,” “normal,” “ordinary,” and “of course” to refer to their uses of mobile phones for initiating contact with their parents. The only exception is the 17-year-old participant, M008. He said his parents more often initiated calls and talks instead of him. He said:

*I sometimes initiated contacts with my parents, but not that much, because I'm now a grown-up, my mind is more on the outside world. It's not like when I was a kid, I always wanted to stick to my parents. Now it's the other way round, they are more concerned about me*.

Most of the respondents who were younger than him were in the age of clinging to their parents and mobile phones facilitated means to do so. Additionally, each age group found age-appropriate means to sticking to their migrant parent(s). Users at different life stage show variance in domestication processes of social media (Matassi et al., 2019). What we add here is within the same life stage, users in different age group found gratifications from the flexible range of affordances available on mobile phones. When a 9-year-old participant's, F004, parents left her 2 years ago, she cried almost every day for parents. Every time she cried, she called her mother, and her mother sang lullaby to comfort her over mobile phone. “*It worked*,” said her grandmother. Now, F004 often called her parents in the evening because her parents were busy working until 9:00 pm. Before 9:00 pm, she found new means to proactively alleviate her longings. She sent microblogging-style text on her QQ^1^ space which her parents could access. She sent stickers, text, and voice messages directly to her parents through the one-on-one messaging function on QQ. In addition, when she missed her parents too much, she said, she used to do video call *via* QQ. “*Because I can see dad and mum. Dad and mum could see me too. I'm happy at sight of them, even though I don't know what to say. When I see them, I just want to smile, to rejoice*.” Video calls lessen the kind of asymmetry in communication (Madianou and Miller, 2011), which is caused by long distance, long time of not seeing each other, and socio-economic differences in transnational migration, and in internal migration as well. Moreover, as seen in this case, video calls lessen the yearnings for parental presence. The sight of parents overjoyed the girl though she was not good at having conversations with them yet. As a result, she relied heavily on very basic functions of mobile phone and apps, which she could initiate and handle, including phone calls, texting, audio messaging, and sending stickers for expressing her yearnings for parental attention.

F0014 in the same age had one more way. “*Inside the mobile phone there're dad and mum*,” she said. She referred to the pictures and videos of her parents stored in the photograph album on her grandmother's mobile phone. The videos were shot when her parents were at home with her. She said she could “see” her dad and mum in this way. “*I often browsed them [pictures and videos]. I miss them. I don't' know if they're tired outside*.” Pictures and videos sustained the happy time together and could be felt and experienced again and again. In such way, the pains of separation are lessened to certain extent and mobile phone becomes an object of emotion in addition to a communication tool. For these pre-teen LBC, mobile phones are not only means of almost daily voice exchanges with their distant parents on phone calls, but also channels of everyday parental pampering and objects of everyday parental attachment, in an easy-to-use way.

Most teenage LBC also reported phone calls and audio messaging as the most used means of communication with distant parent(s) and video calls to a lesser extent. Audio and video communication facilitated migrant parent(s) because they were not good and quick at typing Chinese characters, said M006. He also said his migrant mother called him every other day to ask him about his study. Migrant parent(s) use phone calls to conduct intensive mothering (Madianou and Miller, 2011) or parenting, because of the frequencies that they call and the intensive care they show on calls. However, there is no research on the contributions of LBC on such intensive parenting. This research fills the gap. We found the initiatives and proactiveness of LBC in supporting and promoting distant parenting, especially in terms of audio messaging which they used a lot.

Synchronous audio communication such as phone calls made LBC feel that their distant parent(s) were right beside them which was an assurance for them. But they also preferred asynchronous audio messaging to initiate contact with their distant parent(s) as many times a day as they want and on whatever topic they like. Many teenage respondents mentioned the convenience to send audio messages wherever they were or went, and whatever they were doing. “*The audio version of snapping pictures*,” said F0018. They used audio messages to send their distant parent(s) anything they want to share or express anytime and anywhere without the need to get immediate response. They were satisfied with unilateral live reporting of moments of their lives. For our teenage respondents, intensive audio messaging afforded another kind of intensive parenting: LBC invited their migrant parent(s) to participate in their lives, parts that their distant parent(s) might not consider or concern, and they took the lead in such invitations. They kind of took advantage of asymmetry in communication (Madianou and Miller, 2011) as chances to initiate, activate, and lead communication with parent(s). They used the less obtrusive asynchronous audio messaging to create a space for such communication.

Our respondents showed more complex negotiations with video calls, though they all valued the affordance to see their distant parent(s) which brought them more emotional impact. 14-year-old participant, M006, told he would secretly cry sometimes after video calls with his migrant parents because he missed them too much. Seeing his parents brought out the aggrievance resulted from separation and made him even more aware of the loss out of his parents' absence. Some respondents used video calls as means to solve problems and to achieve virtual accompany of their distant parent(s).

For respondents who were incapable or reluctant to use audio and video means to communicate directly with their distant parent(s) that often, they adopted digital means like cross-platform reposting to attach to their parent(s). M0016 mentioned that he quite often felt running out of topics with his mother in audio messaging, and he then searched and reposted short videos that he thought his mother would like. “*This is means to create topics*,” he said. F0012 said that she often reposted funny videos to her migrant parents. “*Videos that made me laugh, I then want to share with them [parents]. I want them to laugh*.” She also shared with her parents' videos of beautiful scenes that she liked very much. She wanted to share her happiness and amuse her parents. Social media apps combined with other content sharing apps afforded our respondents broadened ranges of topics and content in parent–child communication. Such sharing as communication is not unilateral, but usually mutually made between LBC and their distant parent(s). LBC have more initiatives in such asynchronous exchanges, though.

Searching or selecting appropriate videos for sharing with parent(s) reflect the creative labor of LBC in taking the initiatives in parent–child communication. More creative labor is the self-made pictures or videos, engaging more complicated skills and cross-platform competence. F007 was the best at it among our respondents. She had her own Douyin^2^ account, and she often updated with short videos she made about her daily life and selfies. She also mimicked Douyin influencers to make her videos hit. She shared some of these videos with her migrant parents. In addition, she had specially made videos for her parents and extended family.

*I sometimes take some pictures of local scenery and landscape. I dub music to these pictures and turn them into video. I also add captions. I then send these videos to my parents, or directly to the big family chat group. Everyone has praised me*.

These videos are not only demonstrations of her achievements that she wanted to share with her distant parents but also shared scenes that unite the separated family. She kind of created a virtual hometown for her far-away parents. Now in her eighth year of being left over, the longest among our respondents, F007 was the most relaxed when talking about separation. She said, “*I often send bits and pieces of my life to my parents. We maintain relationship and have not been affected by being separated in two places*.” Mobile maintenance of parent–child relationship, with many initiatives and proactiveness from F007, gave her much confidence to manage separation. She was more competent in using digital affordances than her parents and she used digitalized bits and pieces as invitations for her parents to participate in her life.

Our teenage respondents demonstrated digital competence in exploring the rich variety and multimodality of mobile apps and in combining those apps innovatively to perpetuate connections with distant parent(s). They created various mobile phone practices than the phone call communication on which current parent-LBC literature centers (Madianou and Miller, 2011; To et al., 2020).

### Virtual accompany over mobile phones

Left-behind children's initiatives and proactiveness to promote communication with their migrant parent(s) resonate with their parent(s) and contribute to parent–child collaboration on connection maintenance. All our respondents developed or employed multiple digital means to engage their distant parents, such as rousing their attentions, making communication topics, entertaining their parents, displaying their talents, sharing moment of life, and showing love and care. Their distant parent(s) echoed LBC and tried their best to engage with LBC as well. LBC and their parents were mutual friends on QQ or WeChat^3^, so that they got each other's profiles and updates. Scanning LBC's social media account is assurance for some distant mothers and also means of disciplining the LBC (Chib et al., 2014). Many respondents also formed chat groups with their migrant parent(s) or even with bigger family and had interactions there. Family chat groups create a mediated interactive space for separated LBC families. Besides, some respondents and their migrant parent(s) enriched the mutual engagement between them. M005 sometimes live broadcast his life to his migrant father, who took an office post instead of heavy labor work most other migrant parents did. He said:

*Sometimes my dad would accompany me fishing and playing basketball on mobile phone. It happened when he was free in the office, drinking tea beside the tea table, he would then video call me, watching me playing basketball or fishing while drinking tea. Last time my uncle took me to Luoxian reservoir for fishing. I caught several small Tilapia and I made live streaming the whole process on my mobile phone. He watched the whole process*.

M005 seemed to be used to this kind of video accompany from his father, and distance and spatial differences seem to be bridged for them in those long broadcasting. He did a video call to his father while one of the authors was interviewing him and introducing the author to his father. He shared worthy events to his father while the events were unfolding. His father distantly participated in such unfolding by witnessing or even being a member of the ongoing activities. The practices of simultaneous and ongoing video interaction across temporal and geographic distances *via* keeping Skype video calls on computer turned on for a long time create spaces of transconnectivity for transnational families (King-O'Riain, 2015) and generate substantial feeling of connected co-presence which may lessen the negative effect of migration on left-behind family (Pearce et al., 2013). What is added here is that the video calls are mobile, beyond the walls of rooms and roaming with the embodied phone into the much wider outside world. The co-presence occurs not only in home-setting, but in settings covering the rich physical environments and acquaintanceship along the traces of LBC. Mobile co-presence enriches the settings of co-presence and pulls migrant workers back to hometown. The life world of LBC is integrated into that of their migrant parent(s). Therefore, what holds the connection between separated family members is not just emotion (King-O'Riain, 2015) but also the co-engagement in the unfolding of life events. In M005's story, though, the connectivity is mainly one-way since he live streamed his events to his father instead of *vice versa*.

Several respondents also used video calls and live streaming to show their cooking processes and achievements, the unfolding of more mundane activities, to their distant parents. F0019 was once observed to video call her mother while she finished cooking. She showed the dish to her mother and teased: “*Open your mouth, I'll get a spoon for you to taste*.” She behaved as if she and her distant mother were in the same physical space. Virtual accompany as co-engaging with unfolding event brings excitement and liveliness to our respondents and dissolves the emotional distance brought by long separation and routinized phone calls. F0019 said she felt such video calls showing what she was doing made her and her parents more intimate. The sense of intimacy is created by perceived togetherness from life world integration.

Another means of virtual accompany is LBC and their distant father playing online team games together. M0016 said he and his distant father occasionally played the online game *Honor of Kings* together over the weekend while his father was less occupied with work. He was amazed that his father also played this game and his father ranked in higher division than him. He said he was especially excited when playing games with his father and felt very intimate with his father because his father seemed to be his good brother, pal, and fellow gamer. He could be more casual and relaxed with his father. “*It's more effective than making phone calls, we could also voice chat in the game*,” he said. “*But when work comes, he must quit the game. Work first*,” he said. Though his gaming experience was interrupted by his father's work, M0016 showed understanding and was still content with the changing role of his father from authoritarian disciplinarian (Peng and Wong, 2016) to his equal. He thought that the communication effect was better than in phone calls, *via* which parents display multiple roles in parenting but equals of their LBC.

M0016's father was among the few parents of respondents that accepted the interview. He said:

*Playing game with him is more for the intention to accompany him. After all, I'm not staying at home all year round. He and I usually don't have much to talk about. While playing games together, we can talk about things in common. It's kind of accompany the child*.

The convergence of gaming and mobile technologies is seen with divergent local definitions (Hjorth, 2014) and, in this case, it became creations of common interest, topics, and form of togetherness between migrant father and his left-behind son. Mobile team gaming becomes a window for the father to get into his son's world for a while, temporarily eliminating the feeling of alienation between father and son brought by long separation. The father said that he took this opportunity, when his son opened his heart, to teach his son the value of playing hard, studying hard. He tried to strike a balance between virtually accompanying his son and teaching his son on the importance of study.

A less direct and intensive co-engaging between LBC and their distant parent(s) is sending phone location. M0021's father said he asked his son to send him or his wife phone location if his son left the village because the couple was concerned about the boy's safety. Sending phone location to distant parent(s) facilitates them to keep an eye on their LBC when children are out of the perceived safe distance from home. M0021 said he felt cared about when being required to do so. Location-tracking applications on mobile phones trigger ambiguities of care and control and good or not parenting (Widmer and Albrechtslund, 2021). But in this case, surveillance on LBC's movement out of safety concern by distant parents made the child feel good. Distant location-tracking assures the migrant parents as well.

Virtual accompany as direct or indirect co-engaging with ongoing activities is based on collaborated efforts of LBC and their migrant parent(s). It dissolves not only temporal-spatial barriers out of physical distance between LBC and migrant parent(s) but also emotional estrangement between them because of long separation. However, such dissolving is mostly temporary because it engages parent(s) so much that most our respondents' parents could not afford that much time and attention owing to long working hours.

### Mobile phones: A source of practical solution to problems

F001's family was the most underprivileged in our respondents. Her father suffered from chronic renal failure and needed constant treatment. Her family lived on income from her migrant mother and financial aids from relatives and the village community. Her family got much support from both maternal and paternal relatives, including her second-hand mobile phone from one of her aunts. Mobile phone for this family is basically enabling help hotline. F001 said she needed to be able to contact her mother or relatives at any time because of her father's condition. She gave an example that her father was once in serious situation. She called relatives who took her father to hospital by car. “*Otherwise, I have no idea what to do. The mobile phone is vitally useful*.” Except for affording her much assurance, mobile phone brought her weekly ritual of calls with her distant mother. She was observed to attach huge importance to her mother's call on every weekend afternoon. She fully charged the phone before the call and then waited quietly on the big stone in front of her house for her mother's call. Her younger brother was beside her and asked to speak with their mother from time to time. She asked her mother questions on various aspect of life since she was the backbone of the family: taking care of her father; attending to her younger brother; doing farm work; raising chickens; cooking; and keeping the house. Her mother answered her questions and instructed her on maintaining relationship with relatives and supervising her younger brother. “*She is often like a commander, asking me over the phone to do this and that*,” she said. She was often observed to call her mother in the field while she was coming up with problems at farm work. She was on speakerphone with her mother and did what her mother instructed her to do. She once took pictures of crops to ask her mother if they were mature. Mobile phone for her is not only help hotline for her father, but also for her conducting of everyday life. To save mobile data cost, F001 occasionally used the video call with her mother to let her mother see her father's situation: his complexion, facial expression, health condition etc., so that her mother could ease some worries while working outside, she said.

F001's migrant mother supported the family not only with her job, but with her mobile parenting. She was perpetually available, instructing on first aid, passing on life and farming skills, providing instantaneous support, solving various problems, and being the psychological pillar of her LBC.

Some other respondents also believed that mobile phone communications with their far-away parents helped them solve problems. F007 said many times when she came across difficulties, her parents could help her solve problems over mobile phone. For example, she said, if she lost her way outside the village, or in the near town, her father would ask her to send him her location and pictures of the surrounding environment. “*That way he can direct me home remotely*,” she said. In this case, the distant father solved his daughter's problem by combining his knowledge of hometown with digital technologies.

Another problem-solving that our respondents frequently mentioned is receiving money transfer *via* mobile phones from distant parent(s). F007 said: “*When I run out of money, I immediately send an audio message to them [parents]. Then I immediately receive money via WeChat. This is problem-solving*.” All teenage respondents had similar experience in receiving instant money transfer from their distant parent(s) to recharge school meal IC card, pay for living expenses, mobile phone bills, online shopping, and get pocket money. That mobile money transfer is considered as problem-solving by LBC is because of the immediacy of such transfer. As M008, who was at boarding high school in the near town, stressed, “*Especially for rural children like us, we're not at home in weekdays and we can't get money from home for living expenses. I just send one audio message and my mum from a thousand miles away transfers the money to my WeChat*.” The immediacy of such transfer is not only afforded by WeChat as a mobile payment and transfer app, but more importantly by migrant parents' perpetual availability and constant attention, over mobile phone, to their LBC. Chinese migrant workers are found to gain gratification from using online money transfer, online shopping, and online payment *via* mobile phones in distant parenting because these capabilities induce in them “the feeling that they are participating in their children's lives and increases their parental satisfaction” (Liu and Leung, 2017, p. 942). Study on Somalian migrant mothers show that sending remittances back to family is central to easing their guilt and maternal tensions (Meyers and Rugunanan, 2020). For Latina migrant mothers, money sent back home are as important as letters and phone calls through which they show emotional ties to sustain family connections (Hondagneu-Sotelo and Avila, 1997). Current literature largely focuses on the parents' perspective in terms of mobile fund transfer. Our research provides a LBC's perspective. That is, immediate money transfer largely facilitates LBC's lives, giving them the sense of distant parental problem-solving and helping them cope with some difficulties caused by separation.

However, not all support could be handled over the phone. Then, 14-year-old M006 said he would secretly cry sometimes after video calls with his migrant parents because he missed them too much. “*Especially when I thought about, in the boarding school, when the weather turned cold, other classmates had their parents send quilts to school one after another, whereas I could only endure until the end of the week to go home and fetch the quilt. At the moment I heard their voices on the phone call, I felt very aggrieved*,” he said. Virtual presence and mediated care and support may alleviate the pains of some LBC at some moments, but for other LBC and at some other moments, they may highlight the separation.

Left-behind children and their migrant parents practiced various mobile phone functionalities and affordances to keep perpetually connected out of the need and demand for connections as means to overcome physical separation. Connections and connectivity created by mobile phone communications mutually facilitate, maintain, and strengthen each other.

## Conclusion

This article applies domestication theory to see how LBC use mobile phones to help them address the parent–child separation. The findings show that LBC families actively and collaboratively use multiple mobile phone affordances, mostly convergent on social media platforms like WeChat and QQ, to perpetuate familial connections embedded with contactability anytime anywhere (Green and Haddon, 2009) and connectivity anywhere anyplace (Vanden Abeele et al., 2018). We argue that convergent mobile phone technologies empower both migrant parents and their LBC, instead of empowering the parents only (Madianou and Miller, 2011), by facilitating shared virtual space in which collaborated efforts to overcome separation between migrant parents and their LBC are made. The empowerment of LBC is demonstrated in their innovative practices in adopting mobile phone affordances and in their proactive and initiative uses of mobile phone and apps to engage with their parent(s). All age group LBC are empowered with age-appropriate affordances, that is, pre-teen LBC use simple apps whereas teenage LBC develop more complicated digital competence in combining various apps.

The empowerment of LBC brings them some agencies in handling separation. The agency configures their abilities to initiate contact, highlight their presences on their distant parent(s)' mobile phones, express emotions, share life moments, and seek parental help over long distance and their digital competence in combining affordances cross platforms to practice those abilities. The agency enables them to decide when, how, and how often to communicate with their distant parent(s), even though some communication is unilateral. Some LBC even have the agency to help create virtual hometown on the shared virtue space of LBC families. Besides, the agency gives some LBC efficacy, that is, the feeling of maintaining relationship with parents.

Migrant parent(s) often echo their LBC's agencies. Collaboratively, the mobile phone practices of LBC families help configure rich expressions of familial connections in various forms of contact and communication. The variety of such forms represented as combinations of synchronous, asynchronous, and multimodal mobile affordances convergent on mobile phones are not discussed by current scholarship. We argue that Chinese LBC families' various cross-platform practices of mediated parenting in the shared virtual space, particularly the distant solution of LBC's practical problems by migrant parent(s)' uses of convergent mobile phone technologies, enrich current literature's exploration of distant parenting based on phone call communication, video call, and social media, respectively. Such distant parenting, afforded by convergent digital mobile technologies and based on intensive digital collaboration between LBC and parent(s), partly fulfills normal parenting in Chinese context, which means nourishing, supporting, and raising, blended with emotional and material resources to children (Murphy, 2022).

Distant parenting *via* mobile phone is regularized or even routinized as limited perpetual connectedness between LBC and their migrant parent(s), addressing the parent–child separation to certain extent. The limited perpetual connectedness is afforded by multiple means, as instantaneously and visually as they wish but as constantly and frequently as the socio-economic structure of each LBC household allows. That is, the technologically afforded perpetual availability of being connected between LBC and their migrant parent(s) are restricted by the constraints of the socio-economic conditions of each LBC household. “Work first” value, held as a consensus by migrant parent(s) and their LBC, shapes the equality of parent–child connectedness in the shared virtual space. M005 and his father's practices of long simultaneous and ongoing accompanying outside the home are not common to see in other respondents, because very few migrant parents could afford the time to accompany their LBC over long video calls and some LBC could not afford the cost of mobile data. Technology-afforded connectivity exposes the socio-structural immobility in which the communication between LBC and their migrant parents is contextualized (Thornham and Cruz, 2016; Chen, 2022). Some respondents said their parents worked long hours and were not available for receiving calls until around 9:00 pm; some said their parents had to work night shift; some other said their parents were too busy at work to keeping frequent contact with them. The socio-economic status of migrant parent(s) determines the time, length, and frequency of activating perpetual connectedness and echoing their LBC's initiatives in the shared virtual space. LBC families adopt mobile phones to add temporal, spatial, and substantial richness and depth to configure connections which overcome separation, even though most migrant parent(s) could not afford much time and attention to satisfy their LBC's expectations owing to long working hours and some LBC could not afford much cost on mobile data and installations of Internet access at home.

Besides, there are always moments that connectivity cannot help. As some respondents said, they wished their parents were with them despite the close contact they built with their parents over the mobile phone. They highlighted the problems of separation that cannot be addressed through shared virtual space and limited perpetuate connectedness.

This research focuses on the empowering side of mobile phones in the hands of LBC in rural China. It is the first attempt in this research direction to examine the empowerment of LBC in handling separation issue by exploring their domestication of mobile phones with various practices. It highlights LBC's agency, labor, and subjectivity in alleviating their yearnings for parental attention, support, and accompany *via* their innovative and proactive practices with mobile phones. These findings contribute to better understandings of the dynamic negotiations between LBC families and new ICTs and therefore provide theoretical support for policy development and social work in utilizing new ICTs to address the LBC problems. This research has several limitations. First, the data were generated and collected from a small-stratified sample in a single village in southern Guangxi Autonomous Region. Therefore, the findings should be applied and generalized with caution. Second, though we have made enormous efforts to access parents and caregivers of LBC for interviews and observations, we made less success in this respect. Further research is needed to adopt a bidirectional perspective to explore the narratives of both migrant parent(s) and their LBC on the uses of mobile phones in addressing the separation problem in their families. Bigger samples in other areas are recommended to generate more and wider understanding on the empowering practices of mobile phones by rural LBC.

## Data availability statement

The datasets presented in this article are not readily available because due to the nature of this research, participants of this study did not agree for their data to be shared publicly, so supporting data is not available. Requests to access the datasets should be directed to YL, 243845228@qq.com.

## Ethics statement

Ethical review and approval was not required for the study on human participants in accordance with the local legislation and institutional requirements. Written informed consent to participate in this study was provided by the participants' legal guardian/next of kin.

## Author contributions

JT and YL conceived and designed the study. YL collected the data. KW did the analysis and drafted the manuscript. All authors have approved the final version of the manuscript for publication.

## Conflict of interest

The authors declare that the research was conducted in the absence of any commercial or financial relationships that could be construed as a potential conflict of interest.

## Publisher's note

All claims expressed in this article are solely those of the authors and do not necessarily represent those of their affiliated organizations, or those of the publisher, the editors and the reviewers. Any product that may be evaluated in this article, or claim that may be made by its manufacturer, is not guaranteed or endorsed by the publisher.

## Footnotes

^1^A widely used social networking site most popular among students and young people.

^2^The Chinese version of TikTok.

^3^The most popular social networking site with many other functionalities like online payment, gaming, etc.

## References

[B1] Baym N. (2015). Personal Connections in the Digital Age. Cambridge: Polity.

[B2] Berker T. Hartmann M. Punie Y. Ward K. (2006). Introduction, in Domestication of Media and Technology, eds Berker T. Hartmann M. Punie Y. Ward K. (Maidenhead: Open University Press), 1–17.

[B3] Cai J. Wang Y. Wang F. Lu J. Li L. Zhou X. (2021). The association of parent-child communication with internet addiction in left-behind children in china: a cross-sectional study. Int. J. Pub. Health. 66, 1–7. 10.3389/ijph.2021.630700 34744584 PMC8565268

[B4] Chen H. (2022). The “connected' caregivers: Exploring the interplay of left-behind women's socio-structural immobilities and communicative mobilities in transnational power geometries. J. Comp. Med. Comm. 27, 1–20. 10.1093/jcmc/zmac003

[B5] Cheng J. Sun Y. H. (2014). Depression and anxiety among left-behind children in China: a systematic review. Child 41, 515–523. 10.1111/cch.12221 25495395

[B6] Chib A. Malik S. Aricat R. G. Kadir S. Z. (2014). Migrant mothering and mobile phones: negotiations of transnational identity. Mob. Med. Commun. 2, 73–93. 10.1177/2050157913506007

[B7] China Internet Network Information Center (2016). 2015 Rural Internet Development Research. Available online at: Report https://www3.cnnic.cn/NMediaFile/old_attach/P020170907348967498375.pdf (accessed September 17, 2022).

[B8] China Internet Network Information Center (2022a). The 49^th^ Statistical Report on China's Internet Development. Available online at: https://cnnic.cn/hlwfzyj/hlwtjbg/202202/P020220407403488048001.pdf (accessed June 10, 2022).

[B9] China Internet Network Information Center (2022b). The 50^th^ Statistical Report on China's Internet Development. Available online at: https://www3.cnnic.cn/NMediaFile/2022/0916/MAIN1663313008837KWI7 82STQL.pdf (accessed September 17, 2022).

[B10] Dai Q. Chu R. (2018). Anxiety, happiness and self-esteem of western Chinese left-behind children. Child Abuse Negl. 86, 403–413. 10.1016/j.chiabu.2016.08.002 27568066

[B11] Demirsu I. (2022). Watching them grow: intergenerational video-calling among transnational families in the age of smartphones. Glob. Networks. 22, 119–133. 10.1111/glob.12334

[B12] Fortunati L. Manganelli A. M. Law P. Yang S. (2010). The mobile phone use in Mainland China: Some insights from an exploratory study in Beijing. Telemat. Inform. 27, 404–417. 10.1016/j.tele.2010.04.003

[B13] Gan Y. Greiffenhagen C. Licoppe C. (2020). Orchestrated openings in video calls: Getting young left-behind children to greet their migrant parents. J. Pragmat. 170, 364–380. 10.1016/j.pragma.2020.09.022

[B14] Ge Y. Song L. Clancy R. F. Qin Y. (2019). Studies on left-behind children in China: reviewing paradigm shifts. New Direct. Child Adol. Devel. 163, 115–135. 10.1002/cad.20267 30615250

[B15] Goggin G. Hjorth L. (2014). Introduction: Mobile Media Research—State of the Art, in The Routledge Companion to Mobile Media, eds Goggin G. Hjorth L. (New York, NY and London: Routledge), 1–8.

[B16] Green N. Haddon L. (2009). Mobile Communications: An Introduction to New Media. Oxford and New York: Berg.

[B17] Grošelj D. (2021). Re-domestication of internet technologies: digital exclusion or digital choice? J. Comp. Med. Commun. 26, 422–440. 10.1093/jcmc/zmab017

[B18] Gu X. (2022). Save the children!: governing left-behind children through family in China's great migration. Curr. Socio. Monog. 70, 513–538. 10.1177/0011392120985874

[B19] Guan C. Tang J. Wang M. (2020). The family politics of new media domestication: an ethnographic study of mobile phones' influences on rural adolescents' socialization in a central Chinese town. Asian J. Commun. 30, 1–19. 10.1080/01292986.2019.1683753

[B20] Haddon H. (2006). the contribution of domestication research to in-home computing and media consumption. Inf. Soc. 22, 195–203. 10.1080/01972240600791325

[B21] Haddon H. (2007). Roger silverstone's legacies: domestication. New Med. Soc. 9, 25–32. 10.1177/1461444807075201

[B22] Haddon H. (2011). Domestication analysis, objects of study, and the centrality of technologies in everyday life. Canad. J. Commun. 36, 311–323. 10.22230/cjc.2011v36n2a2322

[B23] Hartmann M. (2006). The triple articulation of ICTS. Media as technological objects, symbolic environments and individual texts, in Domestication of Media and Technology, eds Berker T. Hartmann M. Punie Y. Ward K. (Maidenhead: Open University Press), 80–102.

[B24] Hine C. (2015). Ethnography for the Internet: Embedded, Embodied and Everyday. London: Bloomsbury Publishing.

[B25] Hjorth L. (2014). The game of being mobile one media history of gaming and mobile technologies in Asia-Pacific. Converg. 13, 369–381. 10.1177/1354856507081955

[B26] Hoang L. Yeoh B. S. A. (2012). Sustaining families across transnational spaces: vietnamese migrant parents and their left-behind children. Asian Stud. Rev. 36, 307–325. 10.1080/10357823.2012.711810

[B27] Hondagneu-Sotelo P. Avila E. (1997). I'm here, but i'm there“: the meanings of Latina transnational motherhood. Gend. Soc. 11, 548–571. 10.1177/089124397011005003

[B28] Hu B. Y. Wu H. Winsler A. Fan X. Song Z. (2020). Parent migration and rural preschool children's early academic and social skill trajectories in China: are ‘left-behind' children really left behind? Early Child. Res. Quart. 51, 317–328. 10.1016/j.ecresq.2019.12.011

[B29] Hu C. Mao D.. (2019). Invisible parents and idealized affection: a study on parent-child communication and relationship maintenance of rural left-behind children. Journal. Res. 158, 57–70.

[B30] Hu H. Gao J. Jiang H. Xing P. (2018). A comparative study of unintentional injuries among schooling left-behind, migrant and residential children in China. Int. J. Eq. health. 17, 47–57. 10.1186/s12939-018-0767-3 29685156 PMC5913874

[B31] Jin X. Chen W. Sun I. Liu L. (2020). Physical health, school performance and delinquency: a comparative study of left-behind and non-left-behind children in rural China. Child Abuse Neg. 109, 1–13. 10.1016/j.chiabu.2020.104707 32932062

[B32] King-O'Riain R. (2015). Emotional streaming and transconnectivity: skype and emotion practices in transnational families in Ireland. Glob. Net. 15, 256–273. 10.1111/glob.12072

[B33] Koylu C. Guo D. Kasakoff A. Adams J. W. (2014). Mapping family connectedness across space and time. Cartog. Geog. Infor. Sci. 41, 14–26. 10.1080/15230406.2013.865303

[B34] Lan T. Jia X. Lin D. Liu X. (2019). Stressful life events, depression, and non-suicidal self-injury among Chinese left-behind children: moderating effects of self-esteem. Fron. Psych. 10, 1–11. 10.3389/fpsyt.2019.00244 31057441 PMC6478804

[B35] Leng X. (2022). Digital revolution and rural family income: evidence from China. J. Rural Stud. 94, 336–343. 10.1016/j.jrurstud.2022.07.004

[B36] Li X. Zhu Q. Li W. (2019). An empirical study on the cognitive attitude of rural children using mobile phones (based on the example of children from rural areas in Northern Suzhou). World Media. 4, 5–23. 10.30547/worldofmedia.4.2019.1

[B37] Liang R. Yang Y. Leeuwen K. V. (2022). Mobile phone parenting in work-separated Chinese families with young children left behind: A qualitative inquiry into parenting dimensions and determinants. Child Fam. Soc. Work. 1–13. 10.1111/cfs.12936

[B38] Liu H. Liu L. Jin X. (2020a). The impact of parental remote migration and parent-child relation types on the psychological resilience of rural left-behind children in China. Int. J. Environ. Res. Pub. Health. 17, 5388. 10.3390/ijerph17155388 32726979 PMC7432675

[B39] Liu H. Zhou Z. Fan X. Wang J. Sun H. Shen C.. (2020b). The Influence of left-behind experience on college students' mental health: A cross-sectional comparative study. Int. J. Environ. Res. Pub. Health. 17, 1511–1526. 10.3390/ijerph17051511 32111048 PMC7084344

[B40] Liu P. L. Leung L. (2017). Migrant parenting and mobile phone use: building quality relationships between Chinese migrant workers and their left-behind children. Appl. Res. Qual. Life. 12, 925–946. 10.1007/s11482-016-9498-z

[B41] Liu X. Wu F. Chu W. (2012). Diffusion of mobile telephony in China: drivers and forecasts. IEEE T Eng Manage. 59, 299–309. 10.1109/TEM.2010.2058854

[B42] Liu Y. Erwin L. (2015). Divided motherhood: rural-to-urban migration of married women in contemporary China. J. Comp. Fam. Stud. 46, 241–263. 10.3138/jcfs.46.2.241

[B43] Lu Y. Yeung W. Treiman D. (2020). Parental migration and children's psychological and cognitive development in China: differences and mediating mechanisms. Chinese Socio. Rev. 52, 337–363. 10.1080/21620555.2020.1776600 33767910 PMC7989854

[B44] Lyu Y. Chow J. Hwang J. Li Z. Ren C. Xie J. (2022). Psychological well-being of left-behind children in China: text mining of the social media website Zhihu. Int. J. Environ. Res. Pub. Health. 19, 2127–2140. 10.3390/ijerph19042127 35206315 PMC8871950

[B45] Madianou M. (2014). Polymedia communication and mediatized migration: an ethnographic approach, in Mediatization of Communication, eds K. Lundby (Berlin/Boston: de Gruyter GmbH), 323–346.

[B46] Madianou M. Miller D. (2011). Mobile phone parenting: reconfiguring relationships between Filipina migrant mothers and their left-behind children. New Media Soc. 13, 457–470. 10.1177/1461444810393903

[B47] Martínez C. Olsson T. (2021). Domestication outside of the domestic: shaping technology and child in an educational moral economy. Media Cult. Soc. 43, 480–496. 10.1177/0163443720948011

[B48] Matassi M. Boczkowski P. J. Mitchelstein E. (2019). Domesticating whatsapp: family, friends, work, and study in everyday communication. New Media Soc. 21, 2183–2200. 10.1177/1461444819841890

[B49] Meyers C. Rugunanan P. (2020). Mobile-mediated mothering from a distance: a case study of Somali mothers in Port Elizabeth, South Africa. Int. J. Cult. Studies. 23, 656–673. 10.1177/1367877920926645

[B50] Ministry of Civil Affairs (2018). Chart: Data On Rural Left-Behind Children in 2018. Available online at: https://www.mca.gov.cn/article/gk/tjtb/201809/20180900010882.shtml (accessed June 10, 2022).

[B51] Mobile Phone Penetration in China (2013–2019). Available online at: https://www.statista.com/statistics/233295/forecast-of-mobile-phone-user-penetration-in-china/ (accessed September 16, 2022).

[B52] Moran C. (2022). The connected migrant: a scoping review. Converg. 10.1177/13548565221090480

[B53] Mu G. Hu Y. (2016). Living with Vulnerabilities and Opportunities in a Migration Context: Floating Children and Left-Behind Children in China. Rotterdam: Sense Publishers.

[B54] Murphy R. (2022). What does left behind mean to children living in migratory regions in rural China? Geoforum. 129, 181–190. 10.1016/j.geoforum.2022.01.012

[B55] National Statistical Bureau National Report on Migrant Worker Monitoring Survey (2021). Available online at: http://www.stats.gov.cn/xxgk/sjfb/zxfb2020/202204/t20220429_1830139.html (accessed June 10, 2022).

[B56] Oksman V. Turtiainen J. (2004). Mobile communication as a social stage: meanings of mobile communication in everyday life among teenagers in Finland. New Media Soc 6, 319–339. 10.1177/1461444804042518

[B57] Pajnik M. (2015). Nano-media and connected homeliness. Int. J. Commun. 9, 732–752. Available online at: https://ijoc.org/index.php/ijoc/article/view/2009

[B58] Pan L. Tian F. Lu F. Zhang X. Liu Y. Feng W. et al (2013). An exploration on long-distance communications between left-behind children and their parents in China, in Proceedings of the 2013 Conference on Computer Supported Cooperative Work, 1147–1156.

[B59] Pearce K. Slaker J. Ahmad N. (2013). Transnational families in Armenia and information communication technology use. Int. J. Commun. 7, 2128–2156. Available online at: https://ijoc.org/index.php/ijoc/article/view/1810

[B60] Peng Y. Wong O. M. H. (2013). Diversified transnational mothering via telecommunication: intensive, collaborative, and passive. Gend. Soc. 27, 491–513. 10.1177/0891243212473197

[B61] Peng Y. Wong O. M. H. (2016). Who takes care of my left-behind children? migrant mothers and caregivers. Trans Child Care J Fam Issues. 37, 2021–2044. 10.1177/0192513X15578006

[B62] Rainie L. Wellman B. (2012). Networked: The New Social Operating System. Cambridge and London: The MIT Press.

[B63] Reuver M. Nikou S. Bouwman H. (2016). Domestication of smartphones and mobile applications: a quantitative mixed-method study. Mobile Media Commun. 4, 347–370. 10.1177/2050157916649989

[B64] Seymour J. Walsh J. (2013). Displaying families, migrant families and community connectedness: the application of an emerging concept in family life. J. Comp. Family Stud. 44, 689–698. 10.3138/jcfs.44.6.689

[B65] Shah S. F. A. Hess J. M. Goodkind J. R. (2019). Family separation and the impact of digital technology on the mental health of refugee families in the United States: qualitative study. J. Medical Inter. Res. 21, e14171. 10.2196/14171 31482853 PMC6751097

[B66] Silva A. Shella M. (2014). Introduction, in Mobility and Locative Media: Mobile Communication in Hybrid Spaces, eds Silva A. M (London and New York, NY: Routledge), 1–16.

[B67] Silverstone R. (1994). Television and Everyday Life. London and New York, NY: Routledge.

[B68] Silverstone R. (2006). Domesticating domestication. Reflections on the life of a concept, in Domestication of Media and Technology, eds Berker T. Hartmann M. Punie Y. Ward K. (Maidenhead: Open University Press), 229–248.

[B69] Silverstone R. Hirsch E. Morley D. (1992). Information and communication technologies and the moral economy of the household, in Consuming Technologies: Media and Information in Domestic Spaces, eds Silverstone R. Hirsch E. (London: Routledge), 15–31.

[B70] Tang W. Wang G. Hu T. Dai Q. Xu J. Yang Y.. (2018). Mental health and psychosocial problems among Chinese left-behind children: a cross-sectional comparative study. J. Affect. Disorders. 241, 133–141. 10.1016/j.jad.2018.08.017 30121025

[B71] Telve K. (2019). Family involved or left behind in migration? a family-centred perspective towards Estonia-Finland cross-border commuting. Mobilities 14, 715–729. 10.1080/17450101.2019.1600885

[B72] Thornham H. Cruz E. G. (2016). [Im]mobility in the age of [im]mobile phones: Young NEETs and digital practices. New Media Soc. 19, 1–16. 10.1177/1461444816643430

[B73] To S. Lam C. So Y. (2020). Qualitative study of rural-to-urban migrant chinese mothers' experiences in mother-child interactions and self-evaluation. Appl. Res. Qual. Life. 15, 813–833. 10.1007/s11482-019-9704-x

[B74] Vanden Abeele M. M. P. Wolf R. D. Ling R. (2018). Mobile media and social space: How anytime, anyplace connectivity structures everyday life. Med. Commun. 6, 5–14. 10.17645/mac.v6i2.1399

[B75] Wang Q. Zheng X. (2022). Digital compensation: a study on smartphones and the emotional socialization of left-behind children. J. Mass Commun. Monthly. 3, 37–47. 10.15897/j.cnki.cn51-1046/g2.20211015.003

[B76] Wang Y. (2020). Parent-child role reversal in ICT domestication: media brokering activities and emotional labours of Chinese “study mother” in Singapore. J. Child Media 14, 267–284. 10.1080/17482798.2020.1725900

[B77] Widmer S. Albrechtslund A. (2021). The ambiguities of surveillance as care and control: Struggles in the domestication of location-tracking applications by Danish parents. Nordicom Review 42, 79–93. 10.2478/nor-2021-0042

[B78] Willett R. (2017). Domesticating online games for preteens – discursive fields, everyday gaming, and family life. Children's Geograp. 15, 146–159. 10.1080/14733285.2016.1206194

[B79] Wu N. (2021). The research on policy, practice and countermeasures of the care service system for rural left-behind children in China. J. Edu. Sci. Hunun Normal. Univ. 20, 59–68. 10.19503/j.cnki.1671-6124.2021.05.008

[B80] Xie Z. Fang Y. Mai Y. Zhao J. Zhang X. Zhao J. (2021). The role of alexithymia in childhood trauma and suicide risk: a multi-group comparison between left-behind experience students and no left-behind experience students. Person. Indiv. Diff. 172, 1–6. 10.1016/j.paid.2020.110260

[B81] Xu J. H. (2016). Media discourse on cell phone technology and “left-behind children” in China. Global Media J. Can. Edition 9, 87–102.

[B82] Yu B. Li J. Liu W. Huang S. Cao X. (2022). The effect of left-behind experience and self-esteem on aggressive behavior in young adults in china: a cross-sectional study. J. Interper. Viol. 37. 1049–1075. 10.1177/0886260520922373 32438881

[B83] Zhang M. Zhu C. Nyland C. (2014). The institution of hukou-based social exclusion: a unique institution reshaping the characteristics of contemporary urban China. Int. J. Urban Reg. Res. 38, 1437–1457. 10.1111/j.1468-2427.2012.01185.x

[B84] Zhao H. (2018). A qualitative study on the rights of rural left-behind children in Sichuan Province, China. Child. Youth Serv. Rev. 95, 12–18. 10.1016/j.childyouth.2018.10.029

[B85] Zhen R. Li L. Liu X. Zhou X. (2020). Negative life events, depression, and mobile phone dependency among left-behind adolescents in rural China: an interpersonal perspective. Child. Youth Serv. Rev. 109, 1–8. 10.1016/j.childyouth.2019.104688

[B86] Zhou C. Lv Q. Yang N. Wang F. (2021). Left-behind children, parent-child communication and psychological resilience: a structural equation modeling analysis. Int. J. Environ. Res. Pub. Health 18, 5123–5133. 10.3390/ijerph18105123 34066012 PMC8150701

