# Supplementary material for: The bright side of digitization: Assessing the impact of mobile phone domestication on left-behind children in China's rural migrant families
Source: Front Psychol. 2022 Oct 19;13:1003379. doi: 10.3389/fpsyg.2022.1003379 (PMC9627504; doi:10.3389/fpsyg.2022.1003379)
Supplement: Supplementary file 1 [file Table_1.pdf]

APPENDIX

Code	Age	Gender	School Year	Migrant Parent(s)	Year of Being Left Behind	Household Structure
F001	16	Female	Middle School	Mother	5	Sick father, younger brother
F002	13	Female	Middle School	Mother and Father	3	Uncle's family including their adult son and his wife
M003	12	Male	Primary School	Mother and Father	3	Grandparents, their adult son's family with children
F004	9	Female	Primary School	Mother and Father	2	Grandparents
M005	15	Male	Middle School	Mother and Father	5	Grandparents and their adult daughter
M006	14	Male	Middle School	Mother and Father	3	Grandparents and their adult daughter, elder sister
F007	15	Female	Middle School	Mother and Father	7	Grandparents and their adult son, younger brother
M008	17	Male	High School	Mother and Father	5	Grandmother and cousins
F009	14	Female	Middle School	Father	4	Grandmother and her married youngest son's family, stepmother, baby brother
F0010	13	Female	Middle School	Mother and Father	2	Grandmother

F0011	15	Female	Middle School	Mother and Father	5	Grandmother and her married son’s family
F0012	10	Female	Primary School	Mother and Father	3	Grandparents
M0013	13	Male	Middle School	Mother and Father	5	Grandparents, uncles, cousins
F0014	9	Female	Primary School	Mother and Father	3	Grandparents
M0015	17	Male	High School	Mother and Father	6	Grandmother and her married son’s family with children
M0016	14	Male	Middle School	Mother and Father	3	Grandparents and their two married son’s families, both with children
M0017	14	Male	Middle School	Mother	2	Grandparents and their married son’s family
F0018	15	Female	Middle School	Mother and Father	5	Grandparents and their adult son
F0019	16	Female	High School	Mother and Father	5	Grandparents and cousins
M0020	9	Male	Primary School	Mother and Father	5	Adult elder Sister, elder brother,
M0021	15	Male	Middle School	Mother and Father	4	Grandparents and their daughter
